# Cricoid pressure – A misnomer in pediatric anaesthesia

**DOI:** 10.4103/0974-2700.58647

**Published:** 2010

**Authors:** Ahmed Syed Moied, Jyotishka Pal

**Affiliations:** Department of Anaesthesiology and Critical Care, Jawaharlal Nehru Medical College Hospital, Aligarh Muslim University, Aligarh, Uttar Pradesh, India

**Figure 1:**
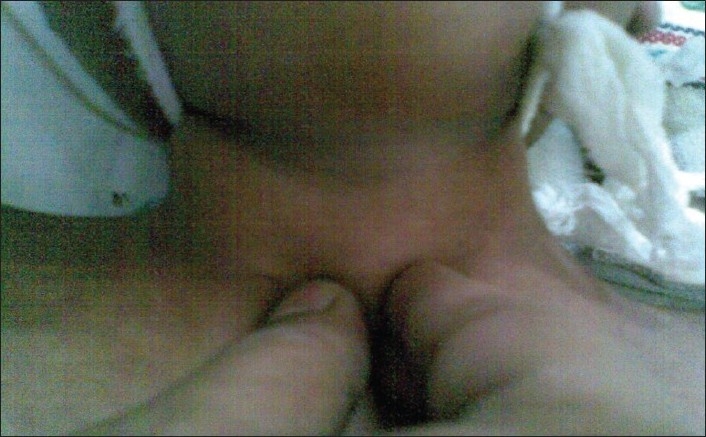
Traditional cricoid pressure

Sir,

Cricoid pressure, sometimes called Sellick's maneuver (or even ‘The Sellicks’), is the application of backward pressure on the cricoid cartilage to occlude the esophagus [Figure 1]. This maneuver prevents aspiration of gastric contents during induction of anesthesia and in resuscitation of emergency victims when intubation is delayed or not possible.

Although the application of cricoid pressure was originally described by Dr. Munro in 1774, it was not until 1961 when Dr. Brian Arthur Sellick, an eminent anesthetist, published his original paper “Cricoid pressure to control regurgitation of stomach contents during induction of anesthesia—preliminary communication” that the maneuver gained widespread acceptance. The recommended pressure to prevent gastric reflux is between 30 and 40 N (equivalent to 3–4 kg). However, pressures higher than 20 N cause pain and retching in awake patients and a pressure of 40 N can distort the larynx and complicate intubation.

Contrary to Arthur Sellick's concept, various authors were of the opinion that cricoid pressure in pediatric population, particularly neonates, improved glottic view and aided tracheal intubation apart from its classical role in rapid sequence intubation for aspiration prophylaxis.[1–6] However, other references quoted differently on the same topic with different and varied nomenclature, for example, pressure over hyoid cartilage, pressure over larynx, pressure over thyroid cartilage, etc.[7–12]

Should the term ‘cricoid pressure’ with the range of pressure and purpose, as per the original article, be applicable in all age groups, especially neonates and pediatrics? Further, does it have a dual purpose when applied to them?

Various modifications have evolved over the manipulation of cricoid with different intent, over the past few years.

‘BURP’ maneuver (consisting of backward, upward, and right-sided pressure on the thyroid and cricoid cartilages) was introduced by Knill in 1993[13] to improve the glottic view during endotracheal intubation. Takahata *et al.* in their study proved the efficacy of BURP by demonstrating significant improvement of the glottic view during the attempts at endotracheal intubation in 630 cases.[15]

In ‘Modified BURP’ maneuver', the patient lies supine with a sniffing position. The thumb and middle finger are applied to the cricoid cartilage and the index finger is applied to the left hand side of the thyroid cartilage. Pressure is applied to both of these structures, downwards, superiorly, and to the right hand side. This maneuver was intended to be a combination of both Sellick's and the burp maneuvers.[13] Snider *et al*. were of the opinion that ‘modified BURP’ maneuver not only failed to enhance the glottic view during RSI but actually worsened it in 30% of cases, and that was because of improper application of cricoid pressure.[13]

**Figure 2:**
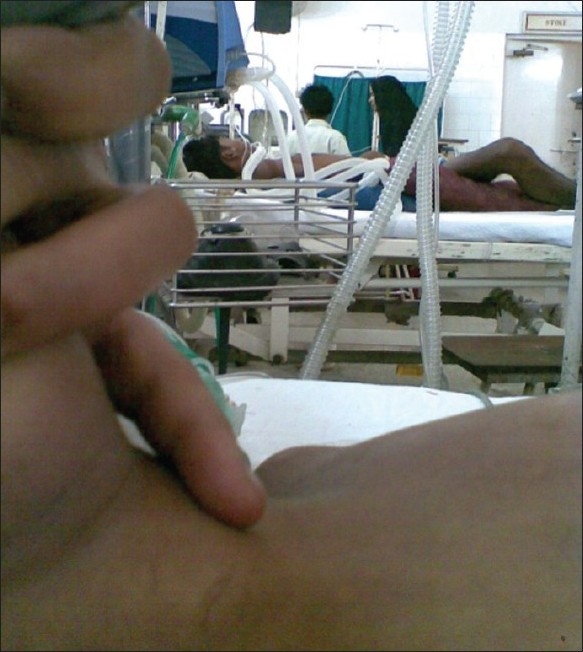
Manipulation of cricoid with little finger

However, understanding the basic purpose of two different maneuvers, the cricoid pressure and the laryngeal manipulation, Benumof coined a new term—OELM (optimal external laryngeal manipulation). He suggested that during laryngoscopy, the operator should manipulate the larynx (hyoid and thyroid cartilages only) with the free hand in an effort to improve the laryngoscopic view [Figure 2]. In a study of 181 patients acting as their own controls, he demonstrated a significant improvement in the laryngoscopic view when OELM was applied.[13]

Hence, confusion, both among practicing anesthesiologists as well as trainee student medics of anesthesia and pediatric medicine, has to arise when one mentions that the most effective maneuver is the application of external pressure at the level of cricoid cartilage to push the larynx into view,[14] whereas another reference quotes that vigorous cricoid pressure can distort the laryngeal anatomy or inadvertently flex the neck, impairing intubation.[6] Further, some say that cricoid pressure prevents regurgitation; others mention that it improves laryngoscopic view.

The concept of the use of ‘cricoid pressure’ was originally intended to prevent aspiration particularly in the setting of emergency intubation. But now, pressure or manipulation of cricoid for whatever and however confusing its purpose may be is being designated the same term of ‘cricoid pressure’. This is the unwarranted jargon, as one specific maneuver intended for a specific purpose is being implemented in other case scenarios with different intent.

To put matters into their right perspective, we opine that apart from its classical role of aspiration prevention, the term ‘cricoid pressure’ should not be used in other scenarios. Rather, a different terminology such as ‘cricoid manipulation’ may be more suitable for external manipulation done over the cricoid for better laryngoscopic view of the glottis, especially in pediatric population.
